# The effect of split application of mineral nitrogen on biomass yield and nitrogen content, uptake, use efficiency and harvest index in spring rye

**DOI:** 10.1038/s41598-025-22332-4

**Published:** 2025-11-03

**Authors:** Andrzej Wysokinski, Stanisław Sienkiewicz, Beata Wiśniewska-Kadżajan, Anna Nogalska, Marcin Becher, Sławomir Józef Krzebietke

**Affiliations:** 1 University of Siedlce, Konarskiego 2 Str., 08110 Siedlce, Poland; 2 University of Warmia and Mazury in Olsztyn, Oczapowskiego 8 Str., 10719 Olsztyn, Poland

**Keywords:** Cereal, Nitrogen, Nitrogen content, Nitrogen doses, Nitrogen harvest index, Nitrogen uptake, Nitrogen use efficiency, Nitrogen utilization efficiency, Yield, Plant sciences, Environmental sciences

## Abstract

The spring form of rye can be grown on soil with relatively low utility value and in areas where winter rye becomes damaged during the winter. Under conditions of soil with low sorption complexes, it is important to adopt the right nitrogen application strategy to achieve high fertilizer efficiency (high yield). This study determined the weight of the separated parts of spring rye, productivity of 1 kg of nitrogen introduced into the soil with mineral fertilizer (E), N content (Nc), N uptake (Nup), N uptake per day (Nup/day), nitrogen use efficiency (NUE), nitrogen utilization efficiency for yield (NutEY), and the nitrogen harvest index (NHI) in six stages of development: BBCH 22, BBCH 33, BBCH 51, BBCH 65, BBCH 75 and BBCH 91. NutEY and NHI were calculated in two ways, with and without including the N accumulated in the roots, and were designated NutEY_1 and NutEY_2 and NHI_1 and NHI_2, respectively. Rye was fertilized once before sowing with 120 kg N ha^−1^ (N1) and with the same amount split into two portions, i.e. 60 + 60 kg N ha^−1^ (N2), and into three portions: 40 + 40 + 40 kg N ha^−1^ (N3). There was also a control treatment without nitrogen application (N0). The first portion was always applied before sowing, and the second and third as top dressing. In most cases, both the stage of development and the nitrogen treatments significantly influenced the values of the spring rye parameters. Split application of nitrogen in two or three portions increased the weight of the whole ears, including the grain, and the whole biomass of spring rye harvested at the fully ripe stage, without significantly altering the weight of the roots or straw. At this development stage, split application of nitrogen did not significantly affect the Nc value in any of the separated parts of the plant. Following the application of nitrogen in three portions, the NutEY_2 values were lower than after application in two portions, while values for NutEY_1, NHI_1, NHI_2, grain yield, whole biomass of spring rye, and Nc and NUE for the grain and whole plant were similar, and the Nup value was lower. On average for the entire growing period, the E value for the grain were not significantly altered by splitting nitrogen application into two or three portions, but the values for the whole spring rye biomass were higher following N application in two or three portions than after a single application. During the growth and development of spring rye, there was an increase in total biomass, total Nup, and NUE, and a decrease in average Nc in the whole plant and in Nup/day. In most cases, the inclusion of the roots in the calculation of NutEY and NHI did not result in statistical differences compared to their calculation without the roots.

## Introduction

Rye cultivation is most common in Northern and Eastern Europe^1^. Research data indicate that the grain of this cereal is an important material for the production of healthy food, and the potential for developing new products is increasing together with knowledge regarding its transformation during milling and baking^2^. In other parts of the world, it is grown on a smaller scale^1^, mainly as a fodder ingredient and for alcohol production^3^. Winter rye grain contains from 7.8 to 12.9% protein and from 58.5 to 65.3% starch and can be a good addition to fodder or a material for flour production^4,5^. Low soil requirements compared to other cereals and high resistance to soil acidification are the main features of rye, thanks to which the species tolerates sowing on soils of low utility values^6^, on which other cereals have less chance of producing high yields. It is a species resistant to biotic and abiotic stresses^7^, which is of great importance given restrictions on the use of plant protection products and climate change. Thanks to the genetic improvement of the quality traits of rye, it gains the potential to expand as a climate-resistant crop^8^. The area of rye cultivation in the world, in Europe, and in Poland in 2021 amounted to 4.305, 3.526 and 0.762 million ha, respectively^1^. Its winter form is grown most often, while spring rye is less popular. Owing to breeding work conducted in Poland in recent years, the registry of cultivars recommended for cultivation contains eight spring rye cultivars, while previously there was only one^9^. It should also be stressed that it is being cultivated more often (on a larger area) than in previous years. The spring form of rye can be grown following precursor crops harvested in autumn, after which it is not possible to prepare the soil and sow winter rye. Moreover, it can be grown in areas where the form sown in autumn is exposed to extremely unfavourable climate conditions in winter in which winter form cannot overwinter.

To exploit the yield potential of any species, it must be provided with an adequate amount of nutrients during each stage of development. One of the most important nutrients for plants is nitrogen^10,11^. A good supply of this macronutrient to plants promotes a faster rate of photosynthesis by increasing the interception of radiation by the crop and the efficiency of conversion to biomass, thus improving biomass production^12^. Photosynthetic activity is related to the high nitrogen content in leaves^13^.To obtain the correct balance between crop yield and fertilizer efficiency, application of excessive amounts of nitrogen should be avoided^14^.

In agricultural practice, split application of nitrogen into several portions, applied at the right time during cereal growth, is usually more beneficial than a single application before sowing, both in terms of yield and for the environment^15,16^. In the case of synthetic fertilizers, most often the entire pool of nitrogen is available to plants immediately after their application, significantly decreasing in the late phase of their growth^17^. A large amount of nitrogen in the soil at the start of plant growth can lead to substantial losses due to leaching, especially under conditions of light soils and heavy rainfall. Plants in the early stages of development are not able to efficiently utilize large amounts of nitrogen, which can lead to low fertilizer efficiency and excessive growth of the vegetative parts of the plants, increasing the risk of lodging. Split nitrogen application enables more efficient nitrogen utilization by plants, reduces losses of this element, and improves crop quality and yield^18,19^. Especially in wet growing seasons, delaying N application increases the plant’s nitrogen utilization, reducing its losses^20^. Although it requires additional labour, the benefits of better management of nitrogen application usually outweigh the potential difficulties associated with repeatedly going out to the field^16^.

Spring rye has not been studied in terms of nitrogen management throughout its entire growing season. This manuscript presents how dividing the total mineral nitrogen dose affects the formation of biomass of roots, straw, whole ears, including husks and grain, and the whole plant of spring rye, but also on nitrogen management indicators. The novelty of the study is the detailed determination of these indicators in six development stages of spring rye, i.e. in phenological stages from BBCH 22—two branches visible to BBCH 91—full maturity. In addition, the scope of calculations of nitrogen use efficiency (NUE), nitrogen utilization efficiency (NutEY) and nitrogen harvest index (NHI) has been expanded to include N accumulation in the roots, which are most often omitted. Such data have not been available in the literature so far, and this manuscript fills the existing gap. Including nitrogen accumulation in roots in the calculation of spring rye N management indicators takes into account the important aspect of its accumulation in the underground parts of the plant^21^. In popular annual crops, nitrogen accumulated in roots accounts for approximately 14% of total nitrogen uptake^22^.Nitrogen accumulated in both the roots and the vegetative above-ground parts of this cereal may be partially allocated to generative organs. Including nitrogen accumulation in roots in the calculation of N management indicators more accurately describes the amount of nitrogen taken up by the whole plant relative to its allocation to grain yield and to residual N returned to the soil than only taking into account N accumulated in the above-ground parts.

Ammonium sulfate was chosen for fertilization because it introduced sulfur into the soil, which has a beneficial effect on nitrogen metabolism in plants.

The aim of the study was to assess the effect of the stage of development and split application of mineral nitrogen on the content, uptake, utilization, use efficiency, and harvest index of this nutrient by spring rye.

## Methods

### Experimental design

A field experiment was carried out in 2019 in Siedlce, eastern Poland (GPS, N: 52° 17′ 35.9′′, E:22° 30′ 51.3′′, 151 m a.s.l.). It was set up in a two-factorial design in randomized blocks, in triplicate. The first factor (A) was the stage of development at which spring rye was harvested and its features were measured: A1—BBCH 22; A2—BBCH 33; A3—BBCH 51; A4—BBCH 65; A5—BBCH 75; A6—BBCH 91. The second factor (N) was nitrogen application and its division according to the following design: N0—no nitrogen application (control); N1—single pre-sowing application of 120 kg N ha^−1^; N2—total application of 120 kg N ha^−1^ split into two portions: 60 kg N ha^−1^ before sowing, 60 kg N ha^−1^ as top dressing at BBCH 33; and N3—total application of 120 kg N ha^−1^ split into three portions: 40 kg N ha^−1^ before sowing, 40 kg N ha^−1^ as top dressing at BBCH 22, 40 kg N ha^−1^ as top dressing at BBCH 51.

Nitrogen was introduced to the soil in the form of ammonium sulfate (NH_4_)_2_SO_4_. In this fertilizer, sulfur was also introduced into the soil as an accompanying component in the following amounts: N1—138 kg S ha^−1^; N2—69 kg S ha^−1^ before sowing, 69 kg S ha^−1^ as top dressing; and N3—46 kg S ha^−1^ before sowing, and twice 46 kg S ha^−1^ as top dressing. Top dressing fertilization was carried out after dissolving the portion of fertilizer in 0.5 dm^−3^ of distilled water. On the plots where mineral fertilizer was not applied, top dressing was carried out using distilled water alone. In addition, P and K were applied on each plot before sowing in equal amounts corresponding to 40 kg P ha^−1^ (in the form of Ca(H_2_PO_4_)_2_, 19.2% P) and 150 kg K ha^−1^ (as KCl, 49.8% K).

The precursor crop for spring rye was soybean, and all of its parts, including the roots, were collected to a depth of 0.25 m. The experimental plots had an area of 1 m^2^ (1 × 1 m) and were located in the canopy of the test species. The ‘Bojko’ cultivar of spring rye was sown in the first 10 days of April in the amount of 500 germinating seeds per plot. Bojko is one of eight spring rye varieties registered in Poland. It typically reaches a height of 130–150 cm. This variety is characterized by high disease resistance: powdery mildew, rynchosporium, and stem rust. It is not affected by ergot and can be grown in ecological systems. The grain, with its high protein content and high falling number, can be used for milling, feed, and as a feedstock for biogas plants.

No herbicides, fungicides and insecticides were used, and weeds were removed manually. At each development stage analysed for the study, rye was dug up (collected in its entirety from 1 m^2^) to a depth of 0.25 m, separately from each 1 m^2^ plot. Plants collected at BBCH 22, BBCH 33 and BBCH 51 were divided into the roots and aerial parts (leaves and culms), at BBCH 65 into the roots, culms, and ears, and at BBCH 75 and BBCH 91 into the roots, culms (straw), chaff and grain (the total weight of the chaff and grain was the weight of the whole ears).

The soil Luvisol on which the experiment was carried out consisted of the following fractions: 2.0–0.05 mm—86.3%; 0.05–0.002 mm—9.5%; < 0.002 mm—4.2%. The soil had a slightly acidic reaction; its pH in H_2_0 and 1 mol mol^−3^ KCl was 6.0 and 5.8, respectively. Selected soil properties are presented in Table 1.

**Table 1:** Selected elements content in soil.

Soil properties	Unit	Value
N_total_	G kg^−1^	0.60
C_total_		10.40
S_total_		0.095
P_Egner-Riehm_	mg kg^−1^	32.6
K_Egner-Riehm_		48.4
Mg_Schachtschabel_		20.2

Cumulative monthly rainfall totals and average air temperatures during the spring rye growing period are presented in Table 2.

**Table 2:** Cumulative monthly rainfall and average monthly air temperatures in 2019 year and in multiyears (Institute of Meteorology and Water Management, National Research Institute in Warsaw, Siedlce measurement station, GPS N: 52° 18′ 05.1′′, E:22° 24′ 53.9′′).

Month	Cumulation monthly rainfall [mm]		Average monthly air temperatures [°C]	
	2019	Multiyears (1981–2018)	2019	Multiyears (1981–2018)
March, III	34.3	30.6	2.2	5.1
April, IV	8.9	35.3	8.2	9.4
May, V	113.9	59.3	13.7	13.0
June, VI	28.6	71.3	16.4	21.5
July, VII	40.3	69.9	18.6	18.0
Sum/Average from IV to VII	191.7	235.8	14.2	15.5

From stem elongation to flowering, rye is particularly sensitive to water shortages, with a transpiration rate ranging from 400 to 500^23^. In 2019, rainfall in April was about 30 mm lower than the long-term average, but in May, it was nearly twice as high as the long-term averages. Low soil moisture in the early stage of growth may have favoured the development of the root system, and the abundant rainfall in May provided optimal conditions for the development of spring rye. In June and July, rainfall levels were considerably lower than the long-term average. These conditions (dry spells) are appearing in Poland with increasing frequency. The limited water availability during this period leads to reductions in cereal yields, especially spring forms^23^.

### Crop samplings, measurements and calculations

The following were determined in the soil before the experiment:

- pH in 1 mol dm^−3^ KCl using the potentiometric method;
- total nitrogen content (Nt) and total carbon content (Ct) using the CHN autoanalyzer (automatic CHN analyser with an IDC detector, Series II 2400, Perkin-Elmer, Valencia, CA, USA); total amount of sulphur using the ICP-AES emission spectrometry method (Optima 3200 RL spectrometer, Perkin-Elmer, Waltham, MA, USA) after dry soil mineralization at 500 °C;
- P and K available for plants by Egner-Riehm method;
- Mg available for plants by Schachtschabel method.

The following were determined in spring rye samples:

- dry wieght (dw) of separated spring rye parts [g m^−2^], after drying at 75 °C;
- total nitrogen content in isolated parts of spring rye, using the CHN autoanalyzer (automatic CHN analyser with an IDC detector, Series II 2400, Perkin-Elmer, Valencia, CA, USA).

### Calculations and Statistical analysis

1. The mass of harvested spring rye parts (DW) per 1 hectare [t dw 1 ha^−1^] was calculated by multiplying their mass obtained from 1 m^2^ by the number of 1 m^2^ on the area of 1 ha.
2. Efficiency (productivity) of 1 kg N introduced into soil with fertilizer per one day of vegetation was calculated in the time intervals between the studied development stages and for the amount of nitrogen that was introduced into the soil until the harvest of spring rye in a studied development stage
  $$E=\frac{(DW \cdot n\_N-DW \cdot n\_O) -(DW \cdot p\_N - DW \cdot p\_O)}{Nd}$$
  where: *E*—fertilization efficiency (productivity) of 1kg N [kg dw 1 kg N ha^−1^]; *DW.n_N*—dry weight of separated parts or the whole plant in the next development phase, with nitrogen application [kg dw]; *DW.n_O*—dry weight of separated parts or the whole plant in the next development phase, without nitrogen fertilization [kg dw]; *DW.p_N*—dry weight of separated parts or the whole plant in the preceding development phase, with nitrogen application [kg dw];
  *DW.p_O*—dry weight of separated parts or the whole plant in the preceding development phase, without nitrogen fertilization [kg dw]; *Nd*—the amount of nitrogen introduced into the soil in the tested treatment until the harvest of spring rye in the studied development phase [kg N ha^−1^], for treatment N1 it was 120 kg N ha^−1^ for all development phases; for treatment N2 *Nd* in the individual development phases it was: BBCH 33 = 60 kg N ha^−1^, BBCH 51-91 = 120 kg N ha^−1^, for treatment N3 *Nd* in the individual development phases it was: BBCH 33 = 40 kg N ha^−1^, BBCH 51 = 80 kg N ha^−1^, BBCH 65-91 = 120 kg N ha^−1^.
3. Total accumulation (uptake) (Nup) of nitrogen by spring rye was calculated by multiplying dry weight of the separated part of spring rye in the studied development phase and nitrogen content in them.
4. Nitrogen accumulation (uptake) by spring rye per one day of vegetation:
  $$Nup/day =\frac{Nup\_n-Nup\_p}{Dn}$$
  where: *Nup/day*—nitrogen accumulation (uptake) per 1 day of vegetation [kg N ha^−1^ per 1 day]; *Nup_n*—nitrogen accumulation (uptake) in separated parts or the whole plant in the next development phase [kg N ha^−1^]; *Nup_p*—nitrogen accumulation (uptake) in separated parts or the whole plant in the preceding development phase [kg N ha^−1^]; *Dn*—number of days between harvests in the development stages, these values were respectively: BBCH 22–33 = 10 days, BBCH 33–51 = 9 days, BBCH 51–65 = 15 days, BBCH 65–75 = 15 days, BBCH 75–91 = 20 days.
5. Nitrogen use efficiency (NUE) calculating by the difference method:
  $$NUE=\frac{Nup\_N-Nup\_O}{Nd}$$
  where: *Nup_N*—nitrogen accumulation (uptake) in separated parts of spring rye on treatments with nitrogen applied [kg N ha^−1^]; *Nup_0*—nitrogen accumulation (uptake) in separated parts of spring rye on treatments without nitrogen applied [kg N ha^−1^]; *Nd*—the amount of nitrogen introduced into the soil in the tested treatment until the harvest of spring rye in the studied development phase [kg N ha^−1^]—the same value, as given above for efficiency of 1 kg N introduced into soil with fertilizer calculation [kg N ha^−1^].
6. Nitrogen utilisation efficiency_1 (NutEY_1)^24^—this coefficient is the amount of grain yield per 1 kg of N accumulated in above-ground part of spring rye plant. It was calculated by dividing grain yield of spring rye [kg dw ha^−1^] by sum of nitrogen accumulation (uptake) in spring rye straw, chaff and grain [kg N ha^−1^].
7. Nitrogen utilisation efficiency_2 (NutEY_2)—this coefficient is the amount of grain yield per 1 kg of N accumulated in the whole plant of spring rye (according to the authors of this research). It was calculated by dividing grain yield of spring rye [kg dw ha^−1^] by sum of nitrogen accumulation (uptake) in spring rye roots, straw, chaff and grain (whole rye plant) [kg N ha^−1^].
8. Nitrogen harvest index (NHI_1) in spring rye cultivation^25^—this coefficient is the part (fraction) of N accumulated in above-ground part of plant that ends up in the grain. It was calculated by dividing nitrogen accumulation (uptake) in grain yield of spring rye [kg N ha^−1^] by sum of nitrogen accumulation (uptake) in spring rye straw, chaff and grain [kg N ha^−1^].
9. Nitrogen harvest index (NHI_2) in spring rye cultivation—this coefficient is the part (fraction) of N accumulated in whole plant that ends up in the grain (according to the authors of this research). It was calculated by dividing nitrogen accumulation (uptake) in grain yield of spring rye [kg N ha^−1^] by sum of nitrogen accumulation (uptake) in spring rye roots, straw, chaff and grain (whole rye plant) [kg N ha^−1^].

Analysis of variance of the results was performed using an MS Excel spreadsheet. The effect of the split application of mineral nitrogen and development phase on individual features of spring rye was determined using Fisher’s F-test. LSD_0.05_ values and significance of differences between means for characteristics of spring rye were calculated by Tukey’s test.

Two-factor analysis of variance for obtained data were performed according to the following model:

y_ijp_ = m + a_i_ + b_j_ + ab_ij_ + e_ij_.

where: y_ijp_—the value of the examined characteristic, m—population average, a_i_—the effect of studied growth stage, b_j_—the effect of differentiated fertilization treatments, e_ij_—the random error (numbers).

Except for data including:

- dry weight increase of spring rye per one day of growth,
- efficiency (productivity) of 1 kg of nitrogen introduced into soil, and
- nitrogen accumulation by spring rye per one day of vegetation,

All of them calculeted for period between 79 and 91 BBCH by following model:

y_ij_ = m + a_i_ + e_ij_.

where: y_ij_—the value of the examined characteristic, m—population average, a_i_—the effect of differentiated fertilization treatments, e_ij_—the random error (numbers).

Relationships between selected traits were evaluated by linear correlation analysis (p = 0.05) by the Statistica software, statistics package 13.1.336.0 PL (StatSoft Inc., Tulsa, OK, USA).

## Results

### Dry weight of spring rye

The dry weight of the roots and culms of spring rye increased from BBCH 22 up to BBCH 75 and decreased from BBCH 75 to BBCH 91 (Table 3). The weight of the whole ears of the cereal increased from BBCH 65 to BBCH 91, and the grain yield increased from BBCH 75 to BBCH 91. The weight of the chaff did not change significantly between BBCH 75 and BBCH 91. The whole biomass of spring rye increased from the first stage of development in which measurements were taken to the last, i.e. from BBCH 22 to BBCH 91.

**Table 3:** Dry weight (dw) of spring rye parts and whole mass, t dw ha^−1^.

Growth stages (BBCH)	N fertilization treatments	Parts of plant					Whole mass
		Roots	Straw	Ears*	Chaff	Grain	
22	N0	0.40A	0.37A				0.77A
	N1	0.32A	0.69B				1.01A
	N2	0.39A	0.60AB				0.99A
	N3	0.44A	0.51AB				0.95A
	means	0.39a	0.54a				0.93a
33	N0	0.73A	0.91A				1.64A
	N1	0.77A	1.65C				2.42B
	N2	0.84A	1.38B				2.22B
	N3	0.82A	1.32B				2.14B
	means	0.79b	1.32b				2.11b
51	N0	0.91A	1.39A				2.30A
	N1	1.01AB	2.95D				3.96C
	N2	1.13B	2.58C				3.71BC
	N3	1.15B	2.32B				3.47B
	means	1.05c	2.31c				3.36c
65	N0	0.95A	1.99A	0.78A			3.72A
	N1	1.25B	3.84C	0.94A			6.03B
	N2	1.25B	3.58B	0.94A			5.77B
	N3	1.38B	3.49B	0.92A			5.79B
	means	1.21d	3.22d	0.89a			5.32d
75	N0	1.10A	2.88A	1.24A	0.32	0.92A	5.22A
	N1	1.44B	4.39B	2.24B	0.57	1.67B	8.07B
	N2	1.38B	4.17B	2.52C	0.64	1.88B	8.07B
	N3	1.50B	4.15B	2.49BC	0.66	1.83B	8.14B
	means	1.36e	3.90f.	2.12b	0.55	1.57a	7.38e
91	N0	0.96A	2.60A	1.74A	0.32	1.42A	5.30A
	N1	1.09AB	4.10B	3.41B	0.53	2.88B	8.60B
	N2	1.12B	3.89B	4.00C	0.61	3.39C	9.01C
	N3	1.16B	3.99B	3.93C	0.64	3.29C	9.08C
	means	1.08c	3.65e	3.27c	0.52	2.75b	8.00f.
LSD_0.05_	growth stage	0.09	0.14	0.13	n.s	0.10	0.23
	interaction N fertilization/growth stage	0.16	0.25	0.28	n.s	0.28	0.41

*in the BBCH 75 and BBCH 91 growth stages as the sum of chaff and grain.

N0—control object, N1—a single dose of fertilizer, N2—fertilizer dose divided into 2 parts, N3—fertilizer dose divided into 3 parts.

a, b, c, d, e, f—different lower-case letters in the columns indicate a significant difference of the means values for investigated growth stages; A,B,C—different capital letters in the columns indicate a significant difference of the values for N fertilization treatments investigated in the specific growth stage; n.s, not significant.

At the fully ripe stage (BBCH 91), the weight of the whole ears, grain, and whole spring rye plants was lower following single application of nitrogen before sowing than when it was split into two or three equal portions (Table 3). Grain yield, which is most often the main goal of spring rye cultivation harvested at full maturity, was similar after the application of nitrogen in two and three doses. At this stage of development, the weight of the roots, straw and chaff did not change significantly when nitrogen application was split into two or three portions.

**Fig. 1:**
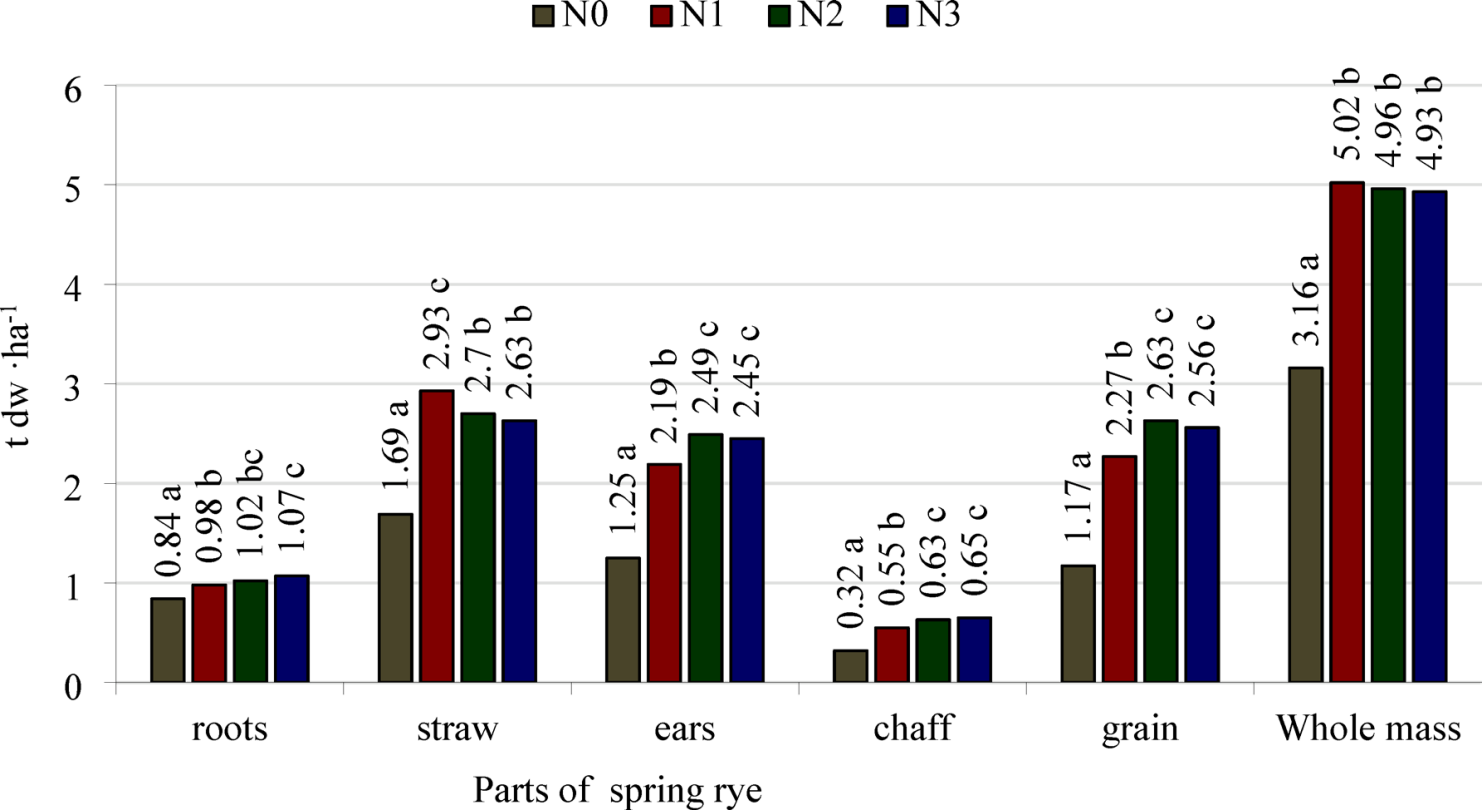
Means values of dry weight (dw) of spring rye parts and whole mass from all investigated growth stages, t dw ha^−1^. N0—control object, N1—a single dose of fertilizer, N2—fertilizer dose divided into 2 parts, N3—fertilizer dose divided into 3 parts. LSD_0.05_: roots = 0.06, straw = 0.10, ears = 0.16, chaff = 0.06, grain = 0.20, and whole mass = 0.17. a, b, c- different lower-case letters next to the values above the bars for the same part of the plant indicate the significant differences between them.

The weight of the roots of spring rye was higher following nitrogen application split into three portions than after a single application of 120 kg ha^−1^ (Fig. 1). In comparison with these treatments, the root weight following nitrogen application split into two portions did not differ significantly. The weight of the whole ears, including the weight of the chaff and the grain, was higher following split application into two or three portions than after a single application of nitrogen before sowing. Culm weight was higher following a single application of nitrogen than after split application in two or three portions. The weight of whole spring rye plants did not change significantly when nitrogen application was split into two or three equal portions. The weights of all separated parts and the whole biomass of spring rye were markedly higher following nitrogen fertilization, irrespective of the number of applications, than in the treatment without application of this nutrient.

The efficiency (productivity) of 1 kg of nitrogen obtained in the intervals between stages of development for all separated parts and the whole biomass of spring rye was not significantly dependent on the splitting of nitrogen application (Table 4). For the whole ears, chaff, grain, and whole spring rye biomass, this parameter was also not significantly dependent on the stage of development. The greatest increase in root weight per kg of nitrogen fertilizer was obtained in the intervals BBCH 22–33, BBCH 33–51, and BBCH 51–65. From BBCH 65, this parameter decreased. In the case of culms/straw, the productivity of 1 kg of nitrogen in the intervals can be ranked in descending order as follows: BBCH 33–51 > BBCH 22–33 = BBCH 51–65 > BBCH 75–91 > BBCH 65–75.

**Table 4:** Efficiency (productivity) of 1 kg of nitrogen introduced into the soil (E), kg dw of spring rye parts and whole mass per 1 kg N.

Period between growth stages (BBCH)	N fertilization treatments	Parts of plant					Whole mass
		Roots	Straw	Ears*	Chaff	Grain	
22–33	N1	1.03	3.41				4.44
	N2	2.20	3.81				6.01
	N3	0.75	3.23				3.98
	Means	1.33c	3.48c				4.81
33–51	N1	0.49	6.88				7.37
	N2	0.83	6.00				6.83
	N3	1.85	6.51				8.36
	Means	1.05c	6.47d				7.52
51–65	N1	1.60	2.45				4.05
	N2	0.62	3.41				4.03
	N3	1.56	4.78				6.34
	Means	1.26c	3.55c				4.81
65–75	N1	0.36	− 2.89	7.10			4.57
	N2	− 0.10	− 2.53	9.37			6.74
	N3	− 0.24	− 1.91	9.28			7.13
	Means	0.01b	− 2.44a	8.58			6.15
75–91	N1	− 1.75	− 0.07	5.46	− 0.36	5.82	3.64
	N2	− 0.99	− 0.01	8.08	− 0.348	8.431	7.08
	N3	− 1.72	1.04	7.77	− 0.23	8.00	7.09
	Means	− 1.49a	0.32b	7.11	− 0.31	7.42	5.94
Averages for N fertilization treatments							
N1		0.35	1.96	6.28			4.82
N2		0.51	2.14	8.73			6.14
N3		0.44	2.73	8.53			6.58
LSD_0.05_	growth stage (A)	0.87	2.71	n.s	–	–	n.s
	N fertilization (N)	n.s	n.s	n.s	n.s	n.s	n.s
	interaction N/A	n.s	n.s	n.s	–	–	n.s

*in the period between BBCH 75 and BBCH 91 growth stages as the sum of chaff and grain.

N1—a single dose of fertilizer, N2—fertilizer dose divided into 2 parts, N3—fertilizer dose divided into 3 parts.

a, b, c, d—different lower-case letters in the columns indicate a significant difference of the means values for investigated growth stages, n.s., not significant.

In the case of the efficiency of 1 kg nitrogen applied to the soil, no significant interaction between the experimental factors was shown (Table 4).

### The content and uptake of nitrogen

**Fig. 2:**
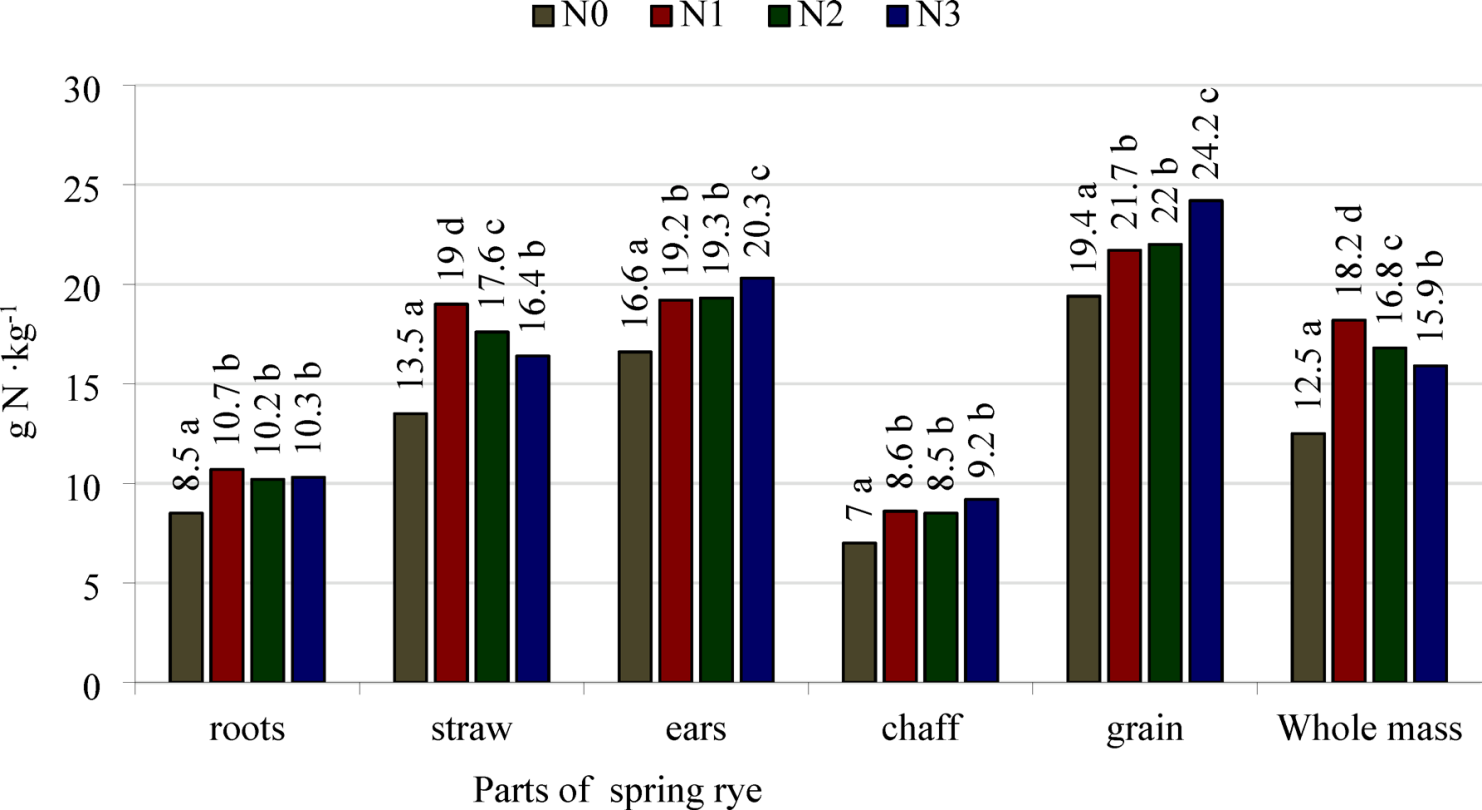
Means values of nitrogen content in spring rye parts and whole mass from all investigated growth stages, g N kg^−1^. N0—control object, N1—a single dose of fertilizer, N2—fertilizer dose divided into 2 parts, N3—fertilizer dose divided into 3 parts. LSD_0.05_: roots = 0.07, straw = 0.08, ears = 0.09, chaff = 0.08, grain = 1.4, and whole mass = 0.05. a, b, c, d—different lower-case letters next to the values above the bars for the same part of the plant indicate the significant differences between them.

The content of nitrogen in the roots, culms/straw, chaff, and grain and on average for the whole spring rye plants was significantly dependent on the stage of development and the nitrogen treatment (Table 5, Fig. 2). Nitrogen content in the roots, culms, and whole plants was highest in the first development stage (BBCH 22) and generally decreased up to BBCH 91. The significance of differences between means was statistically confirmed in all cases except nitrogen content in the roots in BBCH 22 and 33 and in the whole plants in BBCH 75 and 91. In the chaff and grain, the content of nitrogen was lower at BBCH 91 than at BBCH 75. The content of this macronutrient in the ears was similar in BBCH 65, BBCH 75 and BBCH 91.

**Table 5:** Nitrogen content in spring rye parts and whole mass, g N kg^−1^.

Growth stages (BBCH)	N fertilization treatments	Parts of plant					Weighted averages
		Roots	Straw	Ears*	Chaff	Grain	
22	N0	12.6A	30.1A				21.0A
	N1	17.0B	48.8D				38.5D
	N2	13.9A	39.3C				29.3C
	N3	13.2A	36.3B				25.7B
	Means	14.2e	38.6f.				28.6e
33	N0	10.8A	18.5A				15.1A
	N1	15.4C	25.3C				22.1C
	N2	13.6B	24.5BC				20.3BC
	N3	14.1BC	22.8B				19.4B
	Means	13.5e	22.8e				19.3d
51	N0	10.0A	13.2A				12.0A
	N1	12.3B	17.0BC				15.8BC
	N2	13.2B	18.3C				16.8C
	N3	12.8B	16.1B				15.0B
	Means	12.1d	16.2d				14.9c
65	N0	8.4A	10.1A	16.5A			11.0A
	N1	9.5AB	12.1B	19.8B			12.8B
	N2	9.9AB	12.8B	19.3B			13.2B
	N3	10.1B	11.9AB	19.2B			12.6B
	Means	9.5c	11.7c	18.7			12.4b
75	N0	5.4A	5.5A	16.4A	7.0	19.7	8.1A
	N1	6.2AB	6.6A	19.3B	9.3	22.8	10.1B
	N2	6.4AB	6.8A	19.3B	8.8	22.9	10.6BC
	N3	7.4B	7.3A	21.0C	9.4	25.2	11.5C
	Means	6.4b	6.6b	19.0	8.6b	22.6b	10.1a
91	N0	3.8A	3.7A	16.8A	6.9	19.1	8.0A
	N1	3.5A	3.8A	18.6B	8.0	20.5	9.6B
	N2	4.0A	4.0A	19.2B	8.2	21.1	10.7BC
	N3	4.2A	4.1A	20.9C	9.0	23.2	11.4C
	Means	3.9a	3.9a	18.8	8.0a	21.0a	9.9a
LSD_0.05_	Growth stage	0.9	1.0	n.s	0.4	0.7	0.7
	Interaction N fertilization/growth stage	1.6	1.9	1.6	n.s	n.s	1.3

*in the BBCH 75 and BBCH 91 growth stages as the weighted average for chaff and grain.

N0—control object, N1—a single dose of fertilizer, N2—fertilizer dose divided into 2 parts, N3—fertilizer dose divided into 3 parts.

a, b, c, d, e, f—different lower-case letters in the columns indicate a significant difference of the means values for investigated growth stages; A,B,C,D—different capital letters in the columns indicate a significant difference of the values for N fertilization treatments investigated in the specific growth stage; n.s., not significant.

At the fully ripe stage (BBCH 91), the nitrogen content in the roots and straw of spring rye was similar in all N fertilization treatments (Table 5). Its content in the whole ears and on average in the whole plant harvested at BBCH 91 was higher following split application of nitrogen in three portions than after a single application before sowing. The nitrogen concentration in the ears and on average in the whole rye plants fertilized with N split into two portions did not differ significantly compared to single application and split application in three portions.

On average for the growing period, nitrogen content in all separated parts and on average in the whole spring rye plants was lower in the control treatment without nitrogen fertilizer (N0) than following its application in all treatments—N1, N2 and N3 (Fig. 2). Splitting the nitrogen fertilizer into two and three portions had no significant effect on the content of this macronutrient in the roots or chaff. Split application in two portions did not significantly affect the nitrogen content in the whole ears or in the grain, but the N concentration increased in these plant parts following application in three portions. The nitrogen content in the culms/straw and on average in the whole spring rye plant was highest following single application of nitrogen before sowing, lower when it was split into two portions, and the lowest in the case of three portions.

**Fig. 3:**
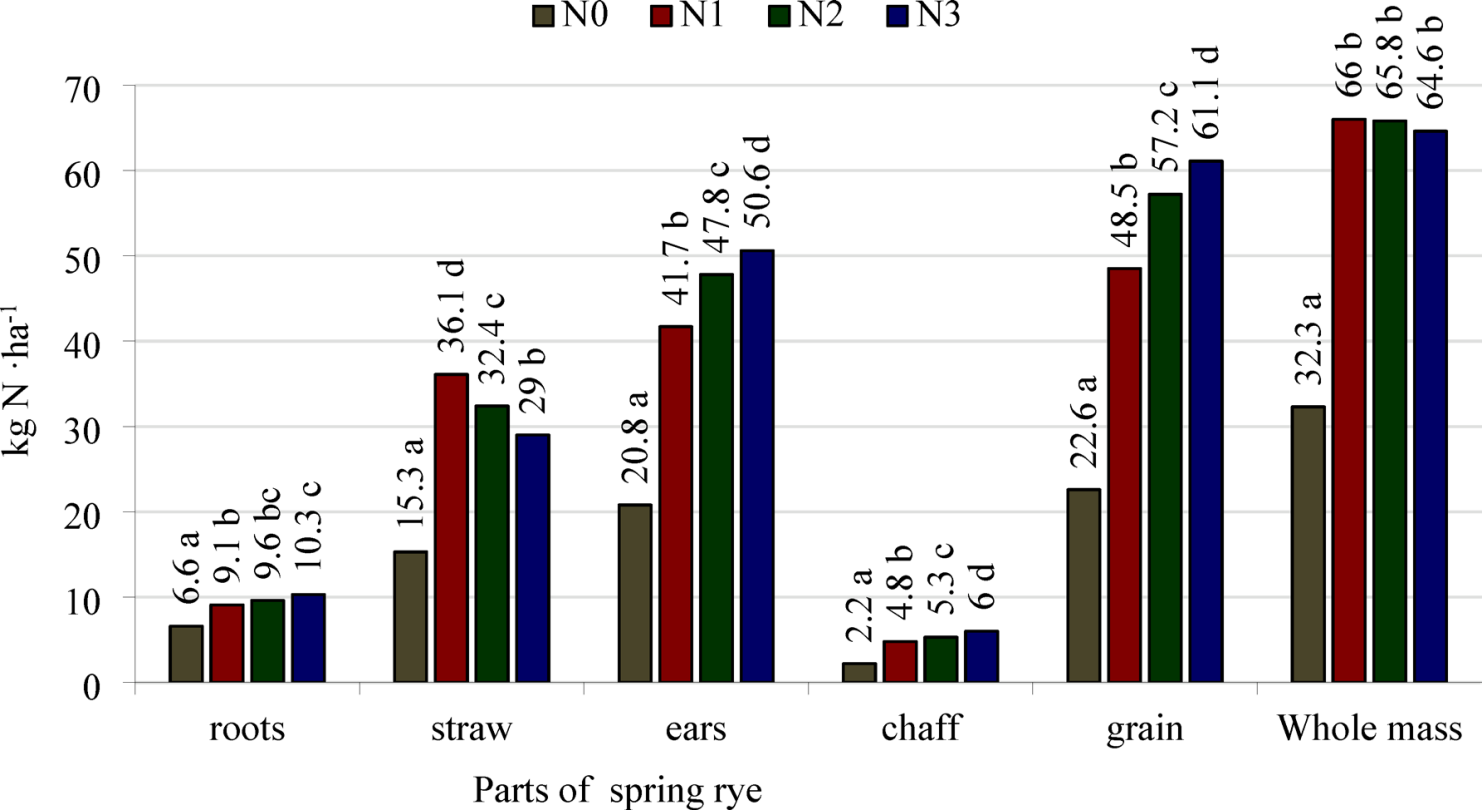
Means values of nitrogen accumulation (uptake) in spring rye parts and whole mass from all investigated growth stages, kg N ha^−1^. N0—control object, N1—a single dose of fertilizer, N2—fertilizer dose divided into 2 parts, N3—fertilizer dose divided into 3 parts. LSD_0.05_: roots = 0.9, straw = 1.2, ears = 2.5, chaff = 0.5, grain = 3.6, and whole mass = 2.2. a, b, c, d—different lower-case letters next to the values above the bars for the same part of the plant indicate the significant differences between them.

The nitrogen accumulation in all separated parts and in the whole biomass of spring rye was significantly dependent on the stage of development and the nitrogen treatment (Table 6, Fig. 3). The amount of nitrogen accumulated in the roots and culms increased from BBCH 22 to BBCH 51. From BBCH stage 51, nitrogen accumulation in the roots decreased up to BBCH stage 91. The amount of nitrogen accumulated in the straw at BBCH 51 and BBCH 65 was similar, but decreased in subsequent stages of development (BBCH 75 and BBCH 91). The amount of nitrogen accumulated in the whole ears increased from BBCH 65 to BBCH 91. Rye accumulated more nitrogen in the grain in BBCH 91 than in BBCH 75, but the pattern was reversed for the chaff. The accumulation of nitrogen in whole spring rye plants increased from the first development stage (BBCH 22) to the last (BBCH 91).

**Table 6:** Nitrogen accumulation (uptake) by spring rye (parts and whole mass), kg N ha^−1^.

Growth stages (BBCH)	N fertilization treatments	Parts of plant					Whole mass
		Roots	Straw	Ears*	Chaff	Grain	
22	N0	5.1A	11.0A				16.1A
	N1	5.5A	33.4D				38.9C
	N2	5.4A	23.5C				28.9B
	N3	5.7A	18.5B				24.2B
	Means	5.4b	21.6b				27.0a
33	N0	7.9A	16.9A				24.8A
	N1	11.9B	41.6D				53.5C
	N2	11.5B	33.7C				45.2B
	N3	11.5B	30.1B				41.6B
	Means	10.7d	30.6d				41.3b
51	N0	9.1A	18.4A				27.5A
	N1	12.6B	50.2D				62.8C
	N2	14.8C	47.2C				62.0C
	N3	14.8C	37.4B				52.2B
	Means	12.8e	38.3e				51.1c
65	N0	8.0A	20.1A	12.8A			40.9A
	N1	12.0B	46.5C	18.5B			77.0B
	N2	12.3B	45.8C	18.2B			76.3B
	N3	14.0B	41.5B	17.6B			73.1B
	Means	11.6d	38.5e	16.7a			66.8d
75	N0	5.9A	15.9A	20.3A	2.2	18.1A	42.1A
	N1	8.9B	28.9B	43.3B	5.3	38.0B	81.1B
	N2	8.8B	28.5B	48.5C	5.7	42.8C	85.8B
	N3	11.1C	30.1B	52.2C	6.2	46.0C	93.4C
	Means	8.7c	25.8c	41.1b	4.9b	36.2a	75.6e
91	N0	3.6A	9.6A	29.3A	2.2	27.1A	42.5A
	N1	3.8A	15.7B	63.2B	4.2	59.0B	82.7B
	N2	4.5A	15.5B	76.6C	5.0	71.6C	96.6C
	N3	4.9A	16.3B	82.0D	5.7	76.3D	103.2D
	Means	4.2c	14.2a	62.8c	4.3a	58.5b	81.2f.
LSD_0.05_	Growth stage	1.2	1.6	2.0	0.3	1.9	3.1
	Interaction N fertilization/growth stage	2.2	2.9	4.4	n.s	4.6	5.5

*in the BBCH 75 and BBCH 91 growth stages as the sum of chaff and grain.

N0—control object, N1—a single dose of fertilizer, N2—fertilizer dose divided into 2 parts, N3—fertilizer dose divided into 3 parts.

a, b, c, d, e, f—different lower-case letters in the columns indicate a significant difference of the means values for investigated growth stages; A,B,C,D—different capital letters in the columns indicate a significant difference of the values for N fertilization treatments investigated in the specific growth stage; n.s., not significant.

At the fully ripe stage (BBCH 91), the amount of nitrogen accumulated in the roots, straw and chaff was similar following a single application of nitrogen and after its application in three portions (Table 6). The amount of nitrogen accumulated in the whole ears, grain, and whole rye plants depending on the nitrogen application treatment can be presented in ascending order as follows: N0 < N1 < N2 < N3.

The amount of nitrogen accumulated in all separated parts and in the whole biomass of spring rye that was not fertilized with nitrogen was lower than in the plants fertilized with this macronutrient (Fig. 3). The amount of nitrogen accumulated in the whole ears and in the grain and the chaff of the cereal fertilized with nitrogen in two portions was higher than after application of the same amount of N in single dose, but it was lower than following N application in three portions. The reverse pattern was noted for the straw. The amount of nitrogen accumulated in the whole rye plants did not differ significantly depending on the number of N applications.

Nitrogen accumulation per day in the roots, straw, and whole plants was highest from BBCH 22 to BBCH 33 and in most cases decreased in the periods between successive stages of development (Table 7). In the periods between BBCH 51 and 60, BBCH 65 and 75, and BBCH 75 and 91, the values for nitrogen accumulation in the roots per day of growth were negative and did not differ significantly. In the case of culms/straw, nitrogen accumulation per day of growth did not differ significantly in the periods between BBCH 22 and 33 and BBCH 33 and 51. In addition, the value of this parameter for straw in the last two periods was negative, which, as in the case of roots, indicates remobilization of this macronutrient to the generative parts. Like the loss of nitrogen from the straw, the rate of N accumulation per day of growth in the whole ears was also higher between BBCH 65 and 75 than between BBCH 75 and 91. The rate of nitrogen accumulation per day of growth in the whole spring rye plants between stages of development can be ranked in descending order as follows: BBCH 22–33 > BBCH 33–51 = BBCH 51–65 > BBCH 65–75 > BBCH 75–91.

**Table 7:** Nitrogen accumulation by spring rye (separated parts and whole mass) per one day of vegetation, kg N ha^−1^ per day.

Period between growth stages (BBCH)	N fertilization treatments	Parts of plant					Whole mass
		Roots	Straw	Ears*	Chaff	Grain	
22–33	N0	0.28A	0.59A				0.87A
	N1	0.64B	0.83AB				1.46AB
	N2	0.61B	1.03B				1.64B
	N3	0.58B	1.15B				1.73B
	means	0.53c	0.90d				1.42d
33–51	N0	0.14AB	0.17A				0.31A
	N1	0.08A	0.96B				1.03B
	N2	0.37B	1.49C				1.86C
	N3	0.36AB	0.82B				1.18B
	means	0.24b	0.86d				1.09c
51–65	N0	− 0.07A	0.11AB				0.89A
	N1	− 0.04A	− 0.25A				0.94A
	N2	− 0.16A	− 0.09AB				0.95A
	N3	− 0.06A	0.27B				1.39A
	means	− 0.08a	0.01c				1.05c
65–75	N0	− 0.14A	− 0.28C	0.50A			0.08A
	N1	− 0.20A	− 1.17A	1.65B			0.28A
	N2	− 0.23A	− 1.15AB	2.02C			0.64A
	N3	− 0.19A	− 0.76B	2.30C			1.35B
	means	− 0.19a	− 0.84a	1.62b			0.59b
75–91	N0	− 0.12A	− 0.32A	0.46A	0.01	0.45	0.02A
	N1	− 0.25A	− 0.66A	0.99B	0.05	0.94	0.08A
	N2	− 0.22A	− 0.65A	1.41C	0.03	1.38	0.54A
	N3	− 0.31A	− 0.69A	1.49C	0.02	1.47	0.49A
	means	− 0.23a	− 0.58b	1.09a	0.03	1.06	0.28a
Averages for N fertilization treatments							
N0		0.02	0.05ab	0.48a			0.43a
N1		0.04	− 0.06a	1.32b			0.76b
N2		0.07	0.12b	1.71c			1.13c
N3		0.08	0.16b	1.90c			1.23c
LSD_0.05_	Growth stage (A)	0.15	0.22	0.12	–	–	0.34
	N fertilization (N)	n.s	0.18	0.23	n.s	0.27	0.28
	Interaction N/A	0.29	0.41	0.32	–	–	0.64

*in the period between BBCH 75 and BBCH 91 growth stages as the sum of chaff and grain.

N0—control object, N1—a single dose of fertilizer, N2—fertilizer dose divided into 2 parts, N3—fertilizer dose divided into 3 parts.

a, b, c, d—different lower-case letters in the columns indicate a significant difference of the means values; for investigated growth stages; A,B,C—different capital letters in the columns indicate a significant difference of the values for N fertilization treatments investigated in the specific growth stage; n.s.—not significant.

The number of nitrogen application procedures did not significantly affect the rate of nitrogen accumulation in the roots or chaff of spring rye (Table 7). Nitrogen accumulation per day of growth in the straw, grain, and whole plants was greater when nitrogen was applied in two or three portions than when it was applied in its entirety before sowing. In the case of straw, ears and whole mass, no significant differences were found in the application of nitrogen in two and three doses. In the case of whole ears and entire plants, the rate of nitrogen accumulation per day was higher in all treatments in which nitrogen was applied (N1, N2 and N3) than in the treatment without nitrogen fertilization (N0).

### Nitrogen management indicators

**Fig. 4:**
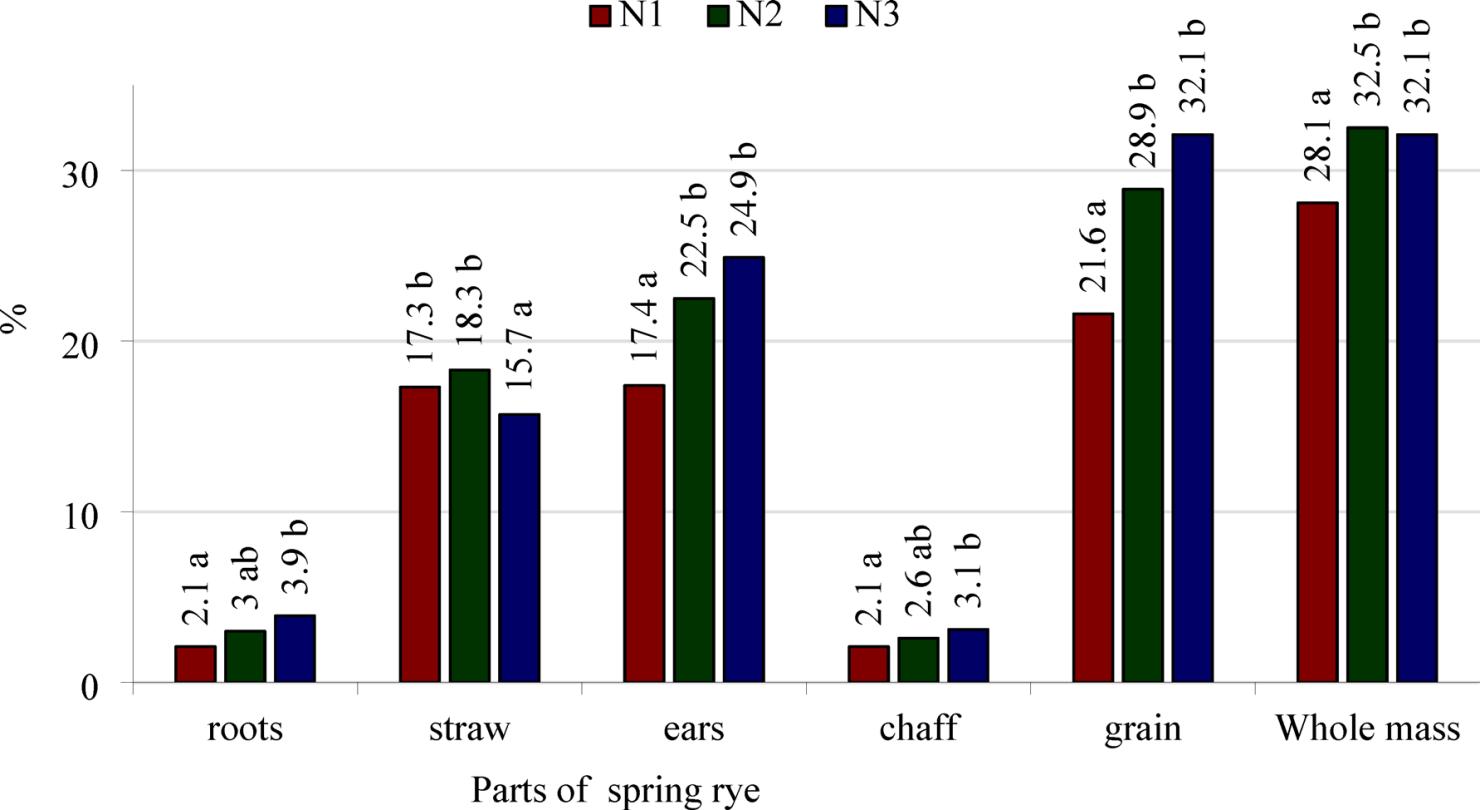
Means values of nitrogen use efficiency (NUE) by spring rye parts and whole mass from all investigated growth stages, kg N ha^−1^. N0—control object, N1—a single dose of fertilizer, N2—fertilizer dose divided into 2 parts, N3—fertilizer dose divided into 3 parts. LSD_0.05_: roots = 1.2, straw = 1.7, ears = 2.7, chaff = 0.6, grain = 3.6, and whole mass = 2.8. a, b—different lower-case letters next to the values above the bars for the same part of the plant indicate the significant differences between them.

Nitrogen use efficiency (NUE) calculated for all separated parts and for the entire spring rye plants was significantly dependent on the stage of development and the nitrogen treatment (Table 8, Fig. 4). The value for this parameter calculated for the roots in stages from BBCH 33 to BBCH 75 did not differ significantly and were higher than at BBCH 22 and BBCH 91. The highest NUE calculated for the culms/straw was obtained during BBCH 51. From BBCH 51, the NUE calculated for straw decreased up to BBCH 91. NUE calculated for whole ears increased from BBCH 65 to BBCH 91. NUE for grain was higher in BBCH 91 than in BBCH 75, while the reverse was noted for the chaff. NUE values calculated for the whole spring rye plants in various stages of development can be presented in ascending order as follows: BBCH 22 < BBCH 33 = BBCH 51 = BBCH 65 < BBCH 75 < BBCH 91.

**Table 8:** Nitrogen use efficiency (NUE) by spring rye calculating by the difference method, %

Growth stages (BBCH)	N fertilization treatments	Parts of plant					Whole mass
		roots	straw	ears*	chaff	grain	
22	N1	0.4	18.6A				19.0A
	N2	0.6	20.7A				21.3A
	N3	1.7	18.7A				20.4A
	means	0.9a	19.3c				20.2a
33	N1	3.4	20.6B				24.0A
	N2	6.0	28.1C				34.1B
	N3	4.6	16.4A				21.0A
	means	4.7b	21.7c				26.4b
51	N1	2.9	26.5A				29.4A
	N2	4.8	23.9A				28.7A
	N3	7.1	23.7A				30.8A
	means	4.9b	24.7d				29.6b
65	N1	3.3	22.0B	4.7A			30.0A
	N2	3.6	21.3AB	4.5A			29.4A
	N3	4.9	17.8A	4.0A			26.7A
	means	3.9b	20.4c	4.4a			28.7b
75	N1	2.5	10.9A	19.2A	2.6	16.6A	32.6A
	N2	2.4	10.5A	23.5AB	2.9	20.6AB	36.4AB
	N3	4.3	11.9A	26.6B	3.3	23.3B	42.8B
	means	3.0b	11.1b	23.1b	2.9b	20.2a	37.2c
91	N1	0.2	5.1A	28.2A	1.7	26.5A	33.5A
	N2	0.7	5.0A	39.4B	2.3	37.1B	45.1B
	N3	1.1	5.6A	43.9B	3.0	40.9B	50.6B
	means	0.6a	5.2a	37.2c	2.3a	34.9b	43.0d
LSD_0.05_	growth stage	2.0	3.0	2.7	0.4	2.4	4.9
	interaction N fertilization /growth stage	n.s	4.2	4.6	n.s	5.1	6.9

*in the BBCH 75 and BBCH 91 growth stages as the sum of chaff and grain.

N1—a single dose of fertilizer, N2—fertilizer dose divided into 2 parts, N3—fertilizer dose divided into 3 parts.

a, b, c, d—different lower-case letters in the columns indicate a significant difference of the means values; for investigated growth stages; A,B,C—different capital letters in the columns indicate a significant difference of the values for N fertilization treatments investigated in the specific growth stage; n.s. —not significant.

At the fully ripe stage (BBCH 91), the NUE values calculated for the roots, straw and chaff were not significantly dependent on the number of nitrogen applications (Table 8). At this stage of development, NUE values calculated for the whole ears, grain and whole rye plants were similar in both treatments with split application of nitrogen and were higher in both cases than for single application.

Splitting nitrogen application into two portions increased the NUE value calculated for the whole ears, grain, and whole spring rye plants without influencing its value for the roots, culms/straw, and chaff (Fig. 4). Splitting nitrogen application into three portions increased the NUE value calculated for whole spring rye plants and all separated parts except straw. In the case of straw, NUE was higher following a single application of nitrogen before sowing than after application split into three portions.

Correlation analysis (p < 0.05) indicates that NUE value was significantly correlated with whole mass of spring rye (r =  + 0.834), total N uptake (r =  + 0.857) and averages N content in whole biomass of tested cereal (r = − 0.829).

**Fig. 5:**
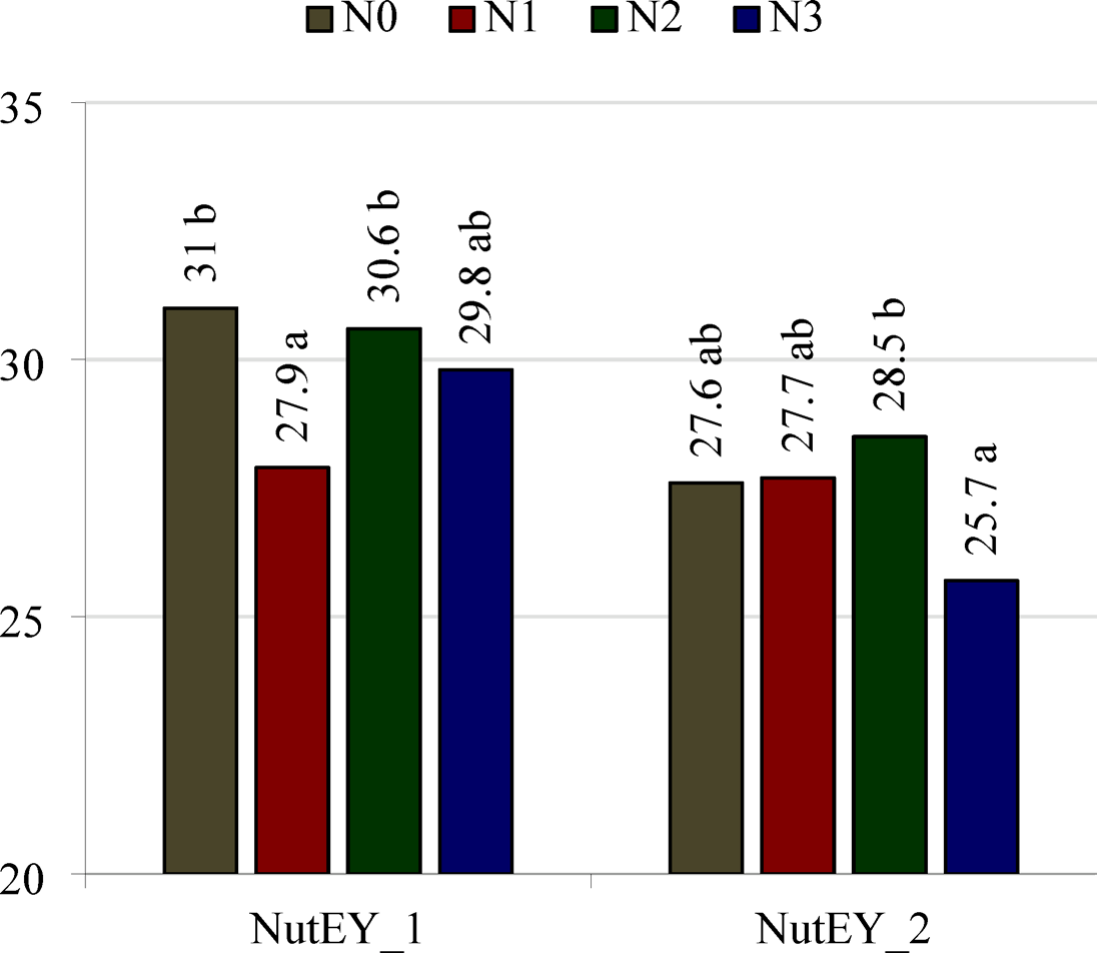
Means values of nitrogen utilisation efficiency (NutEY) of spring rye for 75 and 91 BBCH growth stages. NutEY_1—calculated according to Moll and coauthors^24^, NutEY_2—calculated according to the authors of this research. N0—control object, N1—a single dose of fertilizer, N2—fertilizer dose divided into 2 parts, N3—fertilizer dose divided into 3 parts. LSD_0.05_: NutEY_1 = 2.2, NutEY_2 = 2.1. a, b—different lower-case letters next to the values above the bars for the same part of the plant indicate the significant differences between them.

Nitrogen utilization efficiency for yield (NutEY) calculated according to the formula given by Moll and coauthors^24^, i.e. by dividing the grain yield by total N accumulation in the grain, chaff, and straw (NutEY_1), as well as according to the formula proposed by the authors of this paper, i.e. by dividing the grain yield by total N uptake by the grain, chaff, straw, and roots (NutEY_2), were significantly dependent on the stage of development and the nitrogen treatment (Table 9, Fig. 5). Irrespective how this parameter was calculated, its values were lower at BBCH 75 than at BBCH 91 (Table 9). The highest NutEY_1 values were obtained when spring rye was not fertilized with nitrogen and when the fertilizer was split into two portions, while the lowest value was obtained following single application of nitrogen (Fig. 5). In comparison with these treatments, NutEY_1 obtained in the treatment with split nitrogen application in three portions did not differ significantly. The highest NutEY_2 value was obtained following split application in two portions, and the lowest in the treatment with split application in three portions. In comparison with these treatments, the NutEY_2 values obtained without nitrogen application and following a single application did not differ significantly.

**Table 9:** Nitrogen utilisation efficiency (NutEY) and nitrogen harvest index (NHI) in spring rye cultivation.

Growth stages (BBCH)	N fertilization treatments	NutEY		NHI	
		Calculation methods			
		NutEY_1 [according to Moll and coauthors]^24^	NutEY_2 [according to authors of this research]	NHI_1 [according to Fageria]^25^	NHI_2 [according to authors of this research]
75	N0	25.4	21.8	0.50	0.43
	N1	23.1	20.6	0.53	0.47
	N2	24.4	21.9	0.56	0.50
	N3	22.2	19.6	0.56	0.49
	Means	23.8 a	21.0 a	0.54 a	0.47 a
91	N0	36.6	33.5	0.70	0.64
	N1	36.5	34.8	0.75	0.71
	N2	36.8	35.1	0.78	0.74
	N3	33.5	31.9	0.78	0.74
	Means	35.9 b	33.8 b	0.75 b	0.71 b
LSD_0.05_	Growth stage	1.2	1.1	0.01	0.01
	Interaction N fertilization/growth stage	n.s	n.s	n.s	n.s

N0—control object, N1—a single dose of fertilizer, N2—fertilizer dose divided into 2 parts, N3—fertilizer dose divided into 3 parts.

a, b—different lower-case letters in the columns indicate a significant difference of the means values, n.s., not significant.

Correlation analysis (p = 0.05) indicates that the value of the NutEY_1 and NutEY_2 indices was not significantly correlated with the grain yield of spring rye (r =  + 0.603 and r =  + 0.600, respectively), sum of N uptake by grain, chaff and straw—NutEY_1 (r =  + 0.104) and sum of N uptake by grain, chaff straw and roots—NutEY_2 (r =  + 0.002).

**Fig. 6:**
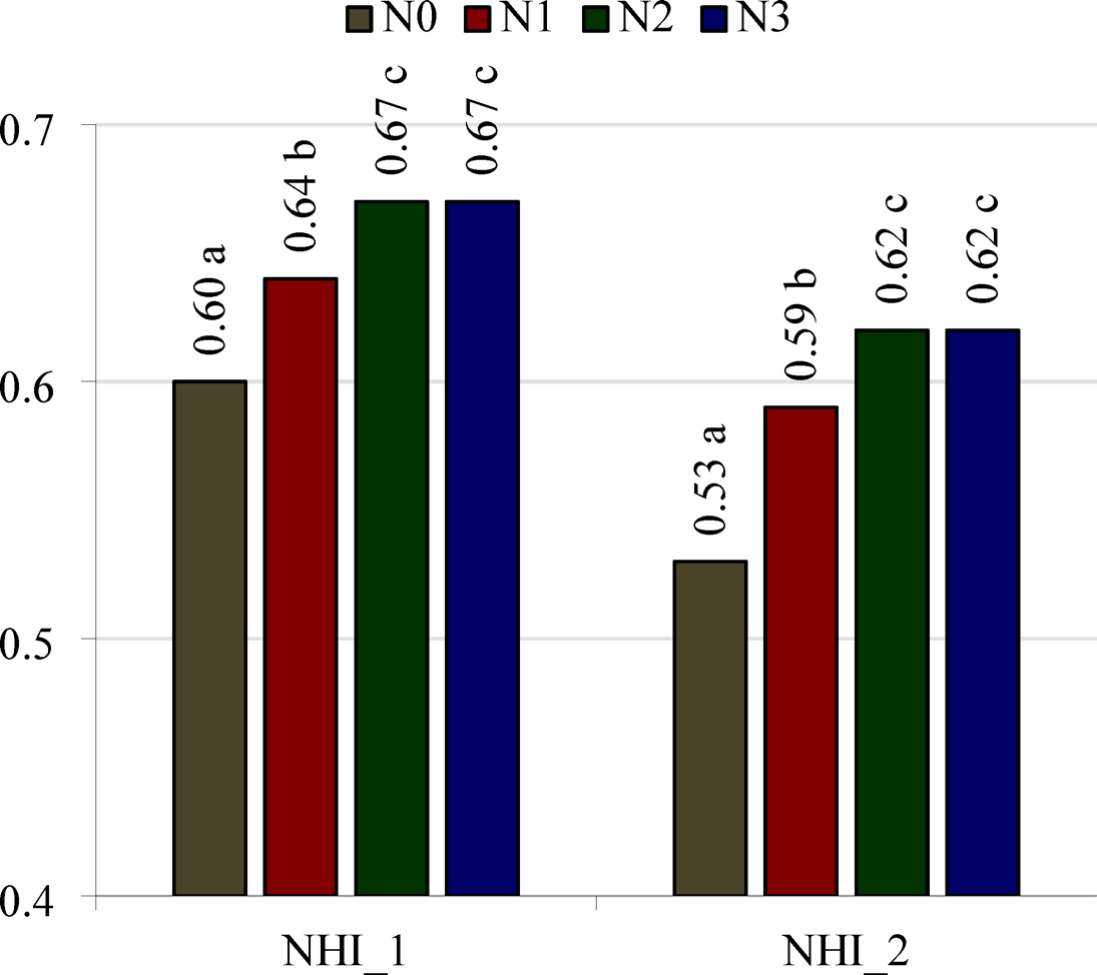
Means values of nitrogen harvest index (NHI) of spring rye for 75 and 91 BBCH growth stages. NHI_1—calculated according to Fageria^25^, NHI_2—calculated according to the authors of this research. N0—control object, N1—a single dose of fertilizer, N2—fertilizer dose divided into 2 parts, N3—fertilizer dose divided into 3 parts. LSD_0.05_: NHI_1 = 0.01, NHI_2 = 0.02. a, b, c—different lower-case letters next to the values above the bars for the same part of the plant indicate the significant differences between them.

The nitrogen harvest index (NHI) calculated according to the formula given by Fageria^25^, i.e. by dividing the N accumulation in the grain by the total N accumulation in the grain, chaff, and straw (NHI_1), as well as according to the formula proposed by the authors of this paper, i.e. by dividing N accumulation in the grain by the total N accumulation in the grain, chaff, straw, and roots (NHI_2), were significantly dependent on the stage of development and the nitrogen treatment (Table 9, Fig. 6). Irrespective of how the parameter was calculated, the values were lower in BBCH 75 than in BBCH 91 (Table 9). The values for both indices (NHI_1 and NHI_2) were lowest when spring rye was not fertilized with nitrogen, higher following single application of nitrogen, and the highest in the case of split application into two or three portions (Fig. 6).

Correlation analysis (p = 0.05) indicates that the value of NHI_1 and NHI_2 coefficients was significantly correlated with the amount of nitrogen accumulated in spring rye grain (r =  + 0.765 and r =  + 0.786, respectively). The value of NHI_1 was not significantly correlated with the sum of N uptake by grain, chaff and straw (r =  + 0.469), and the value of NHI_2 with the sum of N uptake by grain, chaff straw and roots (r =  + 0.399).

## Discussion

### The effect of spliting nitrogen dose on the mass of spring rye

The correct level of nitrogen availability for plants throughout the growing period is beneficial for the photosynthesis rate and accumulation of biomass^26^. Optimal levels of this macronutrient can be supplied to cereals by applying mineral fertilizers^27,28^. Literature data indicate that the application of nitrogen portions at the right stages of development, despite increasing the expenditure of means of production, results in better yields^16^. Other authors draw attention to the need to adjust the timing of nitrogen application to the crop plant and the conditions in which it is grown^29^. In our study in the cultivation of spring rye harvested at the fully ripe stage, this beneficial effect of splitting nitrogen application into two or three portions was observed for the weight of whole ears, including the weight of the chaff and the grain, as well as the weight of the whole plant, but it was not significant for root or straw weight. Rye is usually grown mainly for grain, and the results of our experiment demonstrate that similar yield was obtained irrespective of whether nitrogen was applied in two or three portions. A single early spring nitrogen application did not equal the grain yield effects of split rates but did produce vigorous initial spring vegetative growth. The higher nitrogen content in the blades in the initial development stages of the tested cereal fertilized with the whole dose before sowing than after the application of the divided dose may indicate its greater photosynthetic activity^13^ and the possibility of producing greater vegetative biomass at that time^12^. However, after supplementing nitrogen during the growing season on split-dose treatments, an increase in its content was observed in spring rye blades to levels exceeding that after single-dose treatments, which may also indicate increased photosynthetic activity and increased production of generative organs. The results indicate that in terms of limiting expenditures for fertilizer application, dividing nitrogen fertilizer into two portions is sufficient for spring rye. Applying nitrogen in three doses is justified for a plant with higher requirements, such as wheat. Grain and biomass yields are then highest^16^. In addition, at the fully ripe stage of spring rye, split application of nitrogen in two or three portions produced similar yields of roots, straw, chaff, and thus the whole plant. The size of the root system is important for final yield, because a more extensive root system allows the plant to utilize water and nutrients from the soil more effectively^30,31^. We showed that split nitrogen application into two or three portions did not significantly affect the weight of the roots during the entire growth period or the weight of the culms/straw in the two-tiller, third-node, full flowering, or milky ripe stage. Culm weight from the third-node stage to full flowering was higher following single application of nitrogen than when it was divided into two or three portions. In addition, at the beginning of heading, culm weight was higher following nitrogen application in two portions than in three portions. These observations clearly indicate that the greater nitrogen availability at the start of the growing period initially caused greater development of the above-ground vegetative parts of spring rye without ultimately leading to higher grain yield. This may have been due to the high availability of nitrogen during this period and its reduced availability in the later development stages of rye, as it was dispersed in the environment. Some of the nitrogen which is not taken up by plants can migrate down to the bottom of the soil profile together with rainfall, where it poses a threat to the natural environment^32,33^. The problem of nitrogen migration can be particularly evident in the case of intensive cereal production, in which large amounts of nitrogen fertilizers are applied. Nitrogen application in the later stages of development allows the plant to take up more of this macronutrient during the middle and late growth stages^34^. The experiment showed that splitting the nitrogen fertilizer into two or three portions did not significantly the productivity of 1 kg of nitrogen in the time intervals between the stages of development when the measurements were performed.

### The effect of spliting the nitrogen dose on the N accumulation

Cereals are usually grown for grain, and the remaining parts of the plant are crop residues. Elements accumulated in the grain, including nitrogen, are therefore removed from the field, whereas those contained in the crop residue return to the soil. The experiment showed a positive phenomenon when nitrogen application was split into two and three portions, consisting in an increase in the total amount of nitrogen accumulated in the grain and per day of growth in the time intervals between stages of development. This is related to the higher yield of grain, as evidenced by the significant correlation coefficients (p = 0.05) r =  + 0.870 and r =  + 0.845 respectively, and a slightly higher nitrogen content (r =  + 0.752 and r =  + 0.704 respectively) in these treatments. At the fully ripe stage of spring rye, split application of nitrogen did not significantly modify the uptake of nitrogen by the roots, straw or chaff, and thus the total amount of the element returning to the soil.

Thus, the conversion of nitrogen content in the grain harvested at the full maturity stage of spring rye in into the content of crude protein (CP) (× 6.25) and nitrogen accumulation in the grain into the yield of crude protein (CPY) shows that the use of the fertilizer used in spliting doses caused an increase in both parameters. CP from 12.8% at a single dose increased to 13.2% after spliting it into two parts and up to 15.1% after application it into three parts. CPY from 369 kg ha^−1^ after a single application of the whole dose of fertilizer increased to 447 kg ha^−1^ after dividing it into two parts and up to 477 kg ha^−1^ after spliting it into three parts. The conversion of the average nitrogen accumulation in the spring rye grain obtained in an experiment carried out by Podleśna and coauthors^35^ on the protein yield (343–571 kg ha^−1^) shows, that in the cultivation of this cereal it is possible to get more CPY from area of 1 hectare than in our study.

### The effect of spliting the nitrogen dose on the NUE, NutEY and NHI coefficients

Nitrogen use efficiency (NUE) for the grain and for the whole rye plant was increased when nitrogen was split into two and three portions, from 26.5 to 37.1% and 40.9% for grain and from 33.5 to 45.1% and 50.6% for the whole plant, respectively. However, in all cases, also after dividing the fertilizer dose into three portions, our NUE values were smaller than in the Belete and coauthors^36^ study. These authors, in an experiment with wheat, obtained the highest nitrogen use efficiency (59.7%) following nitrogen application at the same rate as in our experiment (120 kg N ha^−1^), which was divided into three portions (1/4 at sowing, 1/2 at tillering and 1/4 at earing). Bearing in mind the reports that increasing nitrogen doses generally reduces its use efficiency^36,37^, you can see that this parameter is variable and strongly dependent on the conditions in which experiments are conducted. In comparison with another experiment conducted in Poland on spring rye, in which nitrogen applied at doses of 30, 60 and 90 kg N ha^−1^ resulted in NUE values of 25.8%, 35.5% and 30.7%, respectively^35^, the NUE values obtained in the our study can be considered high. Our observations about the beneficial impact of the division of nitrogen dose into parts on its uptake and NUE were also observed in earlier experiments of other researchers with other plants: spring triticale^38^, wheat^36^, and sweet potato^34^. The differences described above NUE values obtained in own study and in experiments of other scientists are large, but researchers testing this parameter in cereal cultivation report that it can range from about 20% to almost 90%^39^.

Split nitrogen application and regular supply of small portions to the plant leads to better nitrogen use efficiency by reducing nitrogen losses into the environment through leaching and denitrification^19^ and to synchronization of the plants’ demand for nitrogen supply, allowing them to take up available nitrogen more efficiently^18^.

Although the NUE index calculated in the present study, which is a ‘fertilizer-based index’, takes into account uptake of available N from soil resources by a plant that has not been fertilized with this macronutrient, its results may nevertheless not be fully accurate, because it does not take into account the plant’s effect on the mobilization of soil nitrogen reserves once available resources have been depleted^21^. Plants have multiple strategies to cope with low nitrogen availability by influencing microbial communities which increase its availability^40^. In this case, there seems to be a need for further research on spring rye using fertilizers enriched with the 15N isotope, which would make it possible to obtain fully accurate results^38,41–43^. Different information is provided by ‘plant-based indices’, which focus on the allocation of plant tissue N towards crop yield or N uptake. These indices can be useful in identifying plant genotypes which are better able to allocate growth or nitrogen resources towards the part of the plant intended for use^21^. Nitrogen utilization efficiency for yield (NutEY) refers to the yield obtained per unit of N uptake by the aerial parts of the plant (NutEY_1)^24^ or, in the modification by the authors of this paper, by the whole plant (NutEY_2). The nitrogen harvest index (NHI) indicates the portion of nitrogen allocated to the crop yield relative to the N accumulated in the aerial parts of the plant (NHI_1)^25^ or, in the modification by the authors of this paper, in the whole plant (NHI_2). In this study, we introduced the additional element in the calculations of NutEY and NHI indices by including the uptake of nitrogen by roots in the calculations because there are considerations in the literature that its omission does not take into account the important aspect of the accumulation of this nutrient in the underground parts of the plant^21^.

The NutEY index can be used to determine the yield produced, e.g. grain yield, per unit of nutrient taken up by the aerial parts of the plant or the whole plant. More productive crops will have higher values for this index. An example of the NutEY value in a maize crop fertilized with nitrogen at rates from 100 to 250 kg ha^−1^ ranged from 40 to 45 kg grain per kg of N uptake^44^. In spring rye, we obtained lower values for this index at the fully ripe stage, averaging 33.8 when N accumulation in the roots was taken into account and 35.9 without the roots. At this stage of development, splitting nitrogen application had no significant effect on this parameter.

The NHI can be used to indirectly determine the amount of nitrogen removed with the crop and the amount that remains and returns to the soil after harvest in the form of crop residue (in the case of cereals, usually the roots, straw and chaff)^21^. Our observations indicate small differences in the value of this coefficient calculated with N accumulation in the roots and without the roots. Their means values were meanly 0.47 and 0.54, respectively, at the milky ripe stage and 0.71 and 0.75 at the fully ripe stage. They indicate that at the fully ripe stage of spring rye, when only the grain is harvested from the field, less than 30% of the nitrogen taken up by the plant returns to the soil. The values for this index at the fully ripe stage of spring rye, irrespective of how it was calculated, were similar in the treatments with single application of N and split application into two and three portions. They amounted to 0.75, 0.78 and 0.78, respectively, when the roots were omitted from the calculations and 0.71, 0.74 and 0.74 when the roots were included. The calculations of the NutEY and NHI indices taking into account nitrogen accumulated in the roots provide a more complete their picture, although it turned out that this did not change the general trends obtained in the study.

The tested yield parameters and nitrogen management in the cultivation of spring rye indicate that the division of nitrogen dose into two parts is sufficient and is the best compromise between slightly increased expenditures for one additional application of fertilizer and the potential production and environmental benefits. Spliting the dose of nitrogen into three parts compared to double application generally does not bring significant changes in the studied parameters, and therefore is not justified. The additional costs incurred for applying the third nitrogen dose would not provide additional production or environmental benefits. A thorough determination of the potential benefits of using divided doses of fertilizer may be difficult due to the variability of production costs, the product produced (grain) and the inability to clearly value environmental benefits.

## Conclusion

Split application of nitrogen into two and three portions did not significantly affect the efficiency (productivity) of 1 kg of nitrogen introduced to the soil. Split nitrogen application into two and three portions increased grain yield, nitrogen accumulation in the whole ears, in the grain, and in the whole spring rye plants harvested at the fully ripe stage, as well as nitrogen uptake per day of growth in these parts and the nitrogen use efficiency value. A single application of nitrogen before sowing led to more extensive development of the aerial parts of spring rye in the initial growth period, ultimately leading to lower grain yield than when nitrogen application was split into two and three portions. Including nitrogen accumulation in the roots in the calculations of NutEY and NHI decreased their values somewhat. Irrespective of how these indices were calculated, at the fully ripe stage of spring rye they were not significantly dependent on division of nitrogen into two and three portions.

To obtain the highest yield of spring rye grain, it is sufficient to split the nitrogen into two portions, one applied before sowing and the other at the stem elongation stage.

## Data Availability

Data is provided within the manuscript file.
